# Maternal factors associated with moderate and severe stunting in Ethiopian children: analysis of some environmental factors based on 2016 demographic health survey

**DOI:** 10.1186/s12937-021-00677-6

**Published:** 2021-02-27

**Authors:** Nebyu Daniel Amaha, Berhanu Teshome Woldeamanuel

**Affiliations:** 1 Department of Nutrition and Dietetics, College of Health Sciences, Mekelle University, Mekelle, Ethiopia; 2 Department of Statistics, College of Natural Sciences, Salale University, Fitche, Oromia Ethiopia

**Keywords:** Undernutrition, Maternal education, Infant and child nutrition, Stunting, Fetal growth, Ethiopia

## Abstract

**Background:**

Stunting or chronic undernutrition is a significant public health problem in Ethiopia. In 2019, 37% of Ethiopian children under-5 were stunted. Stunting results from a complex interaction of individual, household and social (environmental) factors. Improving the mother’s overall care is the most important determinant in reducing the stunting levels in developing countries. We aimed to determine the most important maternal factors associated with stunting and quantify their effects.

**Methods:**

This study used data from the nationally representative 2016 Ethiopian Demographic Health Survey (EDHS). Common maternal factors were first selected and analyzed using Pearson’s chi-square of association followed by multiple logistic regression. To quantify the effect of a unit change of a predictor variable a model for the continuous maternal factors was developed. All analyses were carried out using IBM SPSS© Version 23.

**Results:**

Higher maternal educational level, better maternal autonomy, average or above maternal height and weight, having at least 4 antenatal care (ANC) clinic visits, and delivering in a health facility were significantly associated with lower severe stunting levels. Unemployed mothers were 23% less likely (*p* = 0.003) to have a stunted child compared with employed mothers. Mothers delivering at home had 32% higher odds of stunting (*p* = 0.002). We found that short mothers (< 150 cm) were 2.5 more likely to have stunted children when compared with mothers above 160 cm. Every visit to the ANC clinic reduces stunting odds by 6.8% (*p* <  0.0001). The odds of stunting were reduced by 7% (*p* = 0.028) for every grade a girl spent in school. A unit increase in Body Mass Index (BMI) reduced the odds of stunting by 4% (*p* = 0.014) and every centimeter increase in maternal height reduced the odds of stunting by 0.5% (*p* = 0.01).

**Conclusion:**

Maternal education, number of antenatal care visits, and place of delivery appear to be the most important predictors of child stunting in Ethiopia.. Therefore, educating and empowering women, improving access to family planning and ANC services, and addressing maternal malnutrition are important factors that should be included in policies aiming to reduce childhood stunting in Ethiopia.

## Introduction

Stunting or chronic undernutrition is a condition in which a child is short for his or her age and it affects 22% of all children in the world. Stunting is measured by height-for-age Z (HAZ) score it is classified as moderate stunting (HAZ of less than two standard deviations (SD) below the reference) or severe stunting (HAZ of less than three standard deviations) [1, 2]. Although the global percentage of stunting has decreased from 32.5 to 21.9%, the number of stunted children has increased in Africa from 50.3 million in 2000 to 58.8 million in 2018 [2]. Despite having a very high prevalence of stunted children, Ethiopia has made steady progress in reducing the prevalence of stunting from 58% in 2000 to 37% in 2019 [3–5]. In 2016, 21 and 17% of Ethiopian children under-5 were moderately and severely stunted, respectively. In 2012 the World Health Assembly endorsed the Global Nutrition Targets 2025 which includes a goal of reducing stunting in children under-5 by 40% from its baseline in 2012 [1]. The Ethiopian government is committed to ending child hunger and undernutrition by 2030 and bring stunting in children under 2 years old to zero through its Seqota Declaration [6]. However, the progress made so far has not been enough to achieve the above mentioned goals [7].

Stunting is a complex multifactorial phenomenon that has many contributing factors including poor in utero nutrition, childhood infections, poor maternal health and nutrition, micronutrient deficiencies, poor socioeconomic status, and inadequate infants and young children feeding practices [1, 2, 8]. Pregnant women and young children, in particular, are most vulnerable to inadequate care, access to health services, and sub-optimal feeding practices [6, 9].

Stunting often begins in utero and has been associated with poor breastfeeding and complementary feeding practices; therefore, the time from conception up to 2nd birthday is a critical period during which the detrimental effects of malnutrition could be averted [10]. The 1000-day window period is an important window of opportunity to enhance a child’s nutritional status. A mother is the primary care provider for infants and children. The health and well-being of an infant is to some extent dependent on the mother’s health and well-being. In fact, improvement in maternal care was identified as the most important determinant in reducing stunting levels in Sub-Saharan Africa (SSA) [11]. Previous studies regarding stunting in Ethiopia have found the following factors to be significant predictors of stunting: the age of a child, its gender, size at birth, household wealth, religion, region, place of residence, maternal education, maternal autonomy, maternal height, mother’s BMI, use of contraceptives, maternal mental health, ownership of a mobile phone, and the presence of maternal eye disease [12–20].

If improvements in maternal care are the most important determinants in reducing child stunting as stated above, then determining which of these maternal factors should be prioritized is a crucial step in drafting more enabling intervention policies. The aim of this study is, therefore, to determine the most important maternal factors associated with stunting and quantify the influence of the major factors.

## Methods

### Study population

Nationally representative data were obtained from the Ethiopian Demographic Health Survey (EDHS) cross-sectional study conducted in Ethiopia in 2016 was used [18]. We obtained the data from the DHS MEASURE Program which is available online upon reasonable request. The DHS was conducted by the Central Statistics Agency (CSA) in collaboration with the Federal Ministry of Health and Ethiopian Public Health Institute (EPHI), while the financial support was obtained from the USAID, the government of the Netherlands, the World Bank, Irish Aid, and UNFPA from January 18, 2016 – June 27, 2016. The survey contained information on nutritional status of women and children, maternal and child health, health care use, as well as household socioeconomic variables. Written consent to participate in the study was obtained from adults and/or the parents/guardians of minors.

### Sampling

Ethiopian DHS hired a two-stage stratified cluster design to select respondents for the study. Further, details of the survey design and methodology have been reported in 2016 EDHS [18]. Nationally representative samples of 16,650 households and 15,683 women aged 15–49 years were selected with a response rate of 94.6% from 645 clusters (202 urban areas and 443 rural areas). Probability proportional to enumeration area size and independent selection was employed within each stratum. The anthropometric measurement of 10,552 children was taken [18]. Data on 8855 children aged 6–59 months were considered in this study to identify maternal factors associated with moderate and severe stunting in Ethiopia. Retrospective information about children was obtained from mother’s recall.

### Data collection

The EDHS 2016 used five questionnaires. All the information about the mother was obtained from the “Woman’s Questionnaire” which was used to collect information from all eligible women age 15–49. Weight measurements were obtained using lightweight SECA 878 flat mother-infant scales with a digital screen designed and manufactured under the guidance of UNICEF. Height measurements were carried out using a Shorr® measuring board. Children younger than 24 months were measured for height while lying down, and older children were measured while standing.

### Outcome and predictor variables

The outcome variable in this study was the stunting status of children 6–59 months assessed by using the height-for-age Z (HAZ) scores and classified into not stunted (coded as 0), moderately stunted (coded as 1) or severe stunted (coded as 2). Short for their age HAZ, − 3 SD to − 2 SD was considered moderate and below − 3 SD was considered severely stunted [1].

The explanatory variables included mother’s education (0 = no education, 1 = primary education, 2 = secondary education 0r 3 = higher), mother’s body mass index (BMI) (1 = less than 18.5, 18.5–25 or 3 = greater than 25), number of antenatal care visits before delivery (0 = no visits, 1 = 1–3 visits, 2 = at least four visits), place of delivery (0 = home, 1 = health facility), mother’s height in cm (1 = less than 150 cm, 2 = 150 cm–160 cm, or greater than 160 cm), mother employment status (0 = unemployed, 1 = employed), and mother’s autonomy (measured in their ability to make decisions about how to spend the cash they earn, ownership of a bank account and mobile phones and decision to marry) coded as (1 = low, 2 = middle, 3 = high). These explanatory variables were adopted from various literature based on their theoretical justification [21–26].

### Data analysis and software

Data analyses were performed using the IBM SPSS® Statistics 23.0 statistical software package. Sample weights were used to account for the unequal probability of sampling and non-response. The degree of unadjusted/crude association covariates and the outcome variable was assessed using the chi-square test. The proportional odds model was used as a tool to model child stunting status because the outcome variable (stunting status not stunting, moderate stunting and severe stunting which has a natural ordering) by defining the cumulative probabilities differently rather than considering the probability of a single event [27–30].

The ordinal logistic regression analysis was used since the outcome variable stunting status of children under-5 measured as severe stunting, moderate stunting, and not stunted has a natural order. The main assumption under the proportional odds model was that it is invariant the regression coefficients do not vary when the categories are changed and produce homogeneous estimate overall cut-off points to the effect of predictor variables on the outcome variable. Only the sign changes if the response categories are reversed.

Let Y*i* denote the nutritional status of the *i*^*th*^ child, and the vector X′ = [X_1_, X_2_, …, X_k_] denote a collection of k predictor variables. Then the log-odds of having pr(Y ≤ i) = πi given by:

$$ {\displaystyle \begin{array}{ll}\log it\left(\frac{p\left(Y\le i\right)}{1-p\left(Y\le i\right)}\right)& =\log \left(\frac{\pi_i}{1-{\pi}_i}\right)\\ {}& ={\alpha}_i+{\beta}_1{X}_1+{\beta}_2{X}_2+\dots +{\beta}_k{X}_k\kern1em \end{array}} $$

$$ \mathrm{where}\ {\uppi}_i=\frac{\exp \left({\alpha}_i+{\beta}_1{X}_1+{\beta}_2{X}_2+\dots +{\beta}_k{X}_k\right)}{1+\exp \left({\alpha}_i+{\beta}_1{X}_1+{\beta}_2{X}_2+\dots +{\beta}_k{X}_k\right)} $$

Thus, $\kern0.5em \log it\left[p\left(Y\le i\right)\right]={\alpha}_i+{\sum}_{i=1}^k{X}_i{\beta}_i,i=1,2,\dots, k$ is the proportional odds model for the categorical variable Y, where and β_i_ is a regression coefficient of i^th^ predictor and α_i_ is i^th^ intercept coefficient (threshold value).

The test of parallelism was checked using the likelihood ratio test (LRT). A non-significant test of parallelism was considered an indication that the logit surfaces were parallel. Further, the goodness of fit was done using the Pearson and Deviance tests.

## Results

Majority of mothers (63.7%) had had no education, 25.8% had primary education and 10.5% had secondary or higher level of education, and unemployed (72.2%). About one fourth lower autonomy of decision making and nearly two third (63%) had middle autonomy. More than half (53.4%) of mothers had no ANC visit from skilled provider before delivery. About 66% of the mothers were delivered at home (Table 1).

**Table 1:** Maternal factors associated with moderate and severe stunting in Ethiopia using χ^2^ (chi-square) test

Characteristic	Categories	Number of children	% of children stunted		χ^2^	*p* value
			Moderate	Severe		
		n (%)	n (%)	n (%)		
Mother’s educational level	No education	5645(63.7)	1411(22.5)	1101(19.5)	195.5	< 0.0001
	Primary	2283(25.8)	464(20.3)	331(14.5)		
	Secondary/higher	927(10.5)	130(14.0)	48(5.1)		
Mother’s employment status	Unemployed	6397(72.2)	1260(19.7)	1056(16.5)	0.911	0.634
	Employed	2458(27.8)	507(20.6)	401(16.3)		
Mother’s autonomy	Low autonomy	2109(25.8)	428(20.3)	386(18.3)	9.989	0.041
	Middle autonomy	5274(63.0)	1050(19.9)	839(15.9)		
	High autonomy	982(11.7)	179(18.2)	156(15.9)		
Mother’s BMI^†^(kg/m^2^)	Less than 18.5	2175(24.6)	461(21.2)	426(19.6)	27.95	< 0.0001
	18.5 or higher	6680(75.4)	1303(19.5)	1029(15.4)		
Number of ANC visits before delivery	No ANC	4732(53.4)	923(19.5)	942(19.9)	77.99	< 0.0001
	1–3	1858(21)	372(20.0)	285(15.3)		
	At least 4	2265(25.6)	415(18.3)	245(10.8)		
Place of delivery	Home	5880(66.4)	1211(20.6)	1158(19.7)	158.4	< 0.0001
	Health facility	2975(33.6)	553(18.6)	301(10.1)		
Maternal height (cm)	< 150	1408(15.9)	200(14.2)	244(17.3)	125.46	< 0.0001
	150–160	4667(52.7)	1003(21.5)	779(16.7)		
	> 160	2789(31.5)	438(15.7)	329(11.8)		
Place of residence	Urban	1626(18.4)	296(18.2)	127(7.8)	158.2	< 0.0001
	Rural	7229(81.6)	1605(22.2)	1446(20)		

^†^Body Mass Index, ^‡^ Antenatal Care Clinic * South Nations Nationalities and People Region

The chi-square test was used for testing variation among the proportion of severe and moderate stunting across a set of selected maternal socioeconomic and demographic characteristics. The analysis indicated that mother education level, mother autonomy, mother BMI in kg/m^2^, number of ANC visits before delivery, place of delivery, and mother height in centimeter have a significant association with the stunting status of children. Mother education level showed significant inverse association with both proportion of moderate stunting and severe stunting. Children born to mothers with no education were more likely to moderately stunted (22.5%) and severe stunted (19.5%) as compared to those whose mother with secondary or higher education moderately stunted (14%), and severe stunted (5%).

The proportion of moderate and severe stunting was found to be 20.3 and 18.3% among mothers with low autonomy of decision making, compared to 18.2 and 15.9% among mothers with high autonomy, respectively. Similarly, children to mothers of lower BMI (less than 18.5 kg/m^2^) had the higher proportion of moderate stunting (21.2%), and severe stunting (19.6%). At least four ANC visits were found associated with lower proportion of stunting status, moderate (18.3%) and severe (10.8%) as compared to no ANC visit during pregnancy. Home delivery was more likely associated with the higher moderate and severe stunting than deliveries at health facilities.

The prevalence of stunting was negatively associated with increasing maternal education, maternal autonomy, maternal age at first birth, number of ANC visits, previous birth interval, and maternal height (Table 1). Prevalence of severe stunting was significantly (*p* <  0.0001) lower among mothers with tertiary education, mothers with 1 or 2 children, above 36 months of birth spacing, having at least 4 ANC visits, and the mothers who delivered in a health facility. On the contrary, the prevalence of severe stunting was lower than moderate stunting among mothers who are above 160 cm tall (Table 1).

**Fig. 1:**
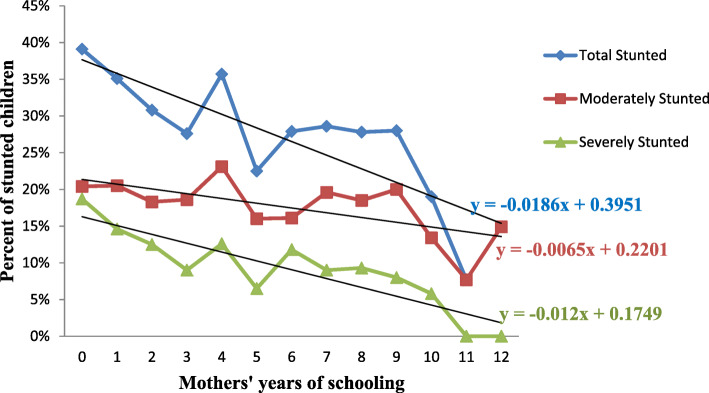
Prevalence of stunting in Ethiopian children under-five declines as maternal educational level increases

The effect of maternal education was further analyzed by plotting the years of maternal education versus the percentage of stunted children (Fig. 1). The figure shows that the prevalence of stunting sharply declines as maternal education increases from grade 1 up to grade 3. Then there is an increase in prevalence from grade 3 to grade 4, and from grade 5 to 6. The total prevalence of stunting plateaus from grade 6 up to grade 10 and then starts to fall sharply. The line of severe stunting starts to flatten out in grade 11 (Fig. 1).

### Maternal predictors of childhood stunting

We assessed the assumption of parallel line using the likelihood ratio test and goodness of fit of the using the Pearson chi-square and Deviance test. According, the likelihood ratio test for testing assumption of parallel line of proportion of odds satisfied, and the Pearson and Deviance test further showed that the is good fit (Table 2).

**Table 2:** Test of goodness of and parallel lines

Tests	Values	*p*-value
LRT	13.15	0.069
Pearson chi-square	433.95	0.027
Deviance	3348	< 0.001

Mothers with primary education were 25% less likely to give birth to stunted children when compared with mothers who had no education (Table 3). Reaching a tertiary level of education reduced the odds by 72% when compared with an uneducated mother. Unemployment was associated with a 23% reduction (OR = 0.768; 95% CI 0.646, 0.912) when compared with employed mothers (Table 3). Underweight mothers were 3.3 times more likely to have a stunted child (*p* = 0.021). The odds of stunting were significantly reduced if a mother had at least 4 ANC visits (OR = 0.763, 95% CI 0.668, 0.870). A mother with short stature (less than 150 cm) was 2.5 times more likely (*p* <  0.0001) to give birth to a stunted child when compared to a mother above 160 cm tall (Table 3).

**Table 3:** Proportional odds model analysis of maternal predictor factors of childhood stunting in Ethiopia, EDHS 2016 given in Odds ratio (OR) and 95% Confidence Interval (CI)

Characteristic	Categories	OR	95% CI	*P* value
Mother educational level	No education	3.316	[1.737, 6.330]	< 0.0001
	Primary	2.619	[1.391, 4.932]	0.003
	Secondary	1.416	[0.583, 3.439]	0.443
	Higher	1		
Mother employment	Unemployed	0.768	[0.646, 0.912]	0.003
	Employed	1		
Mother’ autonomy	High	0.835	[0.703, .992]	0.040
	Middle	0.840	[0.733, .962]	0.012
	Low	1		
Mother’s BMI (kg/m^2^)	Less than 18.5	3.265	[1.178, 9.053]	0.021
	> 25	2.303	[0.876, 6.053]	0.091
	18.5–25	1		
Number of ANC visits before birth	1–3 visits	0.896	[0.804, 0.998]	0.040
	At least 4	0.763	[0.668, 0.870]	< 0.0001
	No ANC	1		
Place of delivery	Home	1.327	[1.113, 1.581]	0.002
	Health facility	1		
Mother’s height (cm)	< 150	2.559	[2.158, 3.035]	< 0.0001
	150–160	1.482	[1.320, 1.664]	< 0.0001
	> 160	1		
Number of ANC visits (per visit)		0.934	[0.913, 0.956]	< 0.0001
Age at first birth (per year)		0.909	[0.857, 0.963]	0.001
Previous birth interval (per month)		0.991	[0.986, 0.997]	0.003
Maternal height (per cm)		0.995	[0.994, 0.996]	0.01
BMI^‡^ in (kg/m^2^) (per unit)		0.959	[0.927, 0.992]	0.014
Maternal education (per year)		0.933	[0.876, 0.993]	0.028
Number of children (per child)		1.094	[1.014, 1.180]	0.041

^†^Body Mass Index, ^‡^ Antenatal Care

The results in Table 3 show that the maternal factors contribute to the odds of child stunting differently. Increasing maternal education years reduced the odds of child stunting by 7% per year, increasing the spacing between births, reduced it by 0.9% per month, 7% reduction was associated with every ANC visit. Moreover, every unit increase of BMI reduced odds of stunting by 4% and every centimeter of maternal height reduced the odds by 1% (Table 3).

## Discussion

This study analyzed the association between childhood stunting and maternal factors that affect stunting using the 2016 EDHS. The most consistent predictors of childhood stunting were low maternal education, short stature, being underweight, not visiting ANC, and delivering at home.

### Education

Maternal education was found to be the strongest predictor of childhood stunting in Ethiopia among the maternal factors studied (Table 1). Educated mothers are more likely to have better health-seeking behavior, practice proper child feeding, and engage in healthy activities during pregnancy and lactation periods. We found that mothers who have no education were 3.3 times more likely to have stunted children when compared with those who had above secondary education. Other studies reported an increased odds of 2.07 in uneducated women in Vietnam [31], 2.02 in Libya [26], 2.26 in Tanzania [21], and 2.53 in Indonesia [32]. The higher odds in our study could be because we compared mothers with no formal education with those who had above secondary education; whereas, the other studies compared uneducated mothers with secondary and/or above combined in one group. However, our estimate of the protective effect of maternal education in Ethiopia was lower than the 6.4 and 4.92 reported by Berhe et al. [33] and Kahsay et al. [34], respectively. This higher odds ratio of stunting in mothers with no formal education could be due to the smaller sample size used Berhe et al. [33] (*n* = 330) and Kahssay et al. [34] (*n* = 322) whereas the EDHS used (*n* = 8855).

Contrary to our findings, having a primary level education did not significantly reduce the odds of stunting in Ghana, whereas having a secondary or higher education significantly reduced it by 44% [24]. This shows that there could be a certain threshold of “school years” for a mother to be in school before the protective effect of maternal education manifests. Reaching 11th grade is associated with the lowest odds of having a severely stunted child in Ethiopia (Fig. 1). A review of childhood stunting in three African countries reported that the threshold for the improvement of stunting was 9 years of schooling in Malawi and 11 years in Tanzania and Zimbabwe [35]. Similar to our finding, maternal education was found to be the most important predictor of stunting in Iran [36]. When quantified by number of years, every year of maternal education was associated with a 7% reduction in the odds of stunting in children (Table 3), this was higher than the 5% per year reduction in Indonesia [37] or the 3% per year reduction in Palestine [38]. In Zambia having a higher education was associated with a 75% reduction in the odds of stunting [39] lower than the 58% reported from the analysis of the 2011 EDHS [20]. This reduction in the odds of stunting could be due to women with higher education are financially more likely to be better off, have enough knowledge on child care, and more likely to follow antenatal and postnatal care.

### Employment

Our analyses found maternal employment is not associated with reduced odds of childhood stunting in this study. Similar with our finding, previous studies in Ethiopia found no association (*p* = 0.634) between employment and stunting [40, 41]. Our hypothesis was that employed mothers would have lower odds of stunting because employment is associated with higher income and standard of living. Nigerian employed mothers had lower odds of having a stunted child [42]. Unemployed mothers had 23% lower odds (OR = 0.768; *p* = 0.003) of having a stunted child than employed mothers (Table 3). In Guatemala, unemployed mothers had 42% lower odds of stunting when compared with employed mothers [43]. There are some possible reasons as to why unemployed mothers have lower odds of stunting. Firstly, stay-at-home mothers (unemployed mothers) are more likely to breastfeed and look after their baby than working mothers and secondly wealthier or “better-off” men in developing countries like Ethiopia don’t usually want their wives to work, therefore the effect of maternal employment could be confounded. Therefore, the effect of maternal employment needs to be considered along with paternal employment and the general household wealth index.

### Height and weight

The weight of a mother is a significant predictor of childhood stunting in Ethiopia (Table 3). In 2016, 22% of Ethiopian women of reproductive age were underweight [16]. Children born to underweight mothers are more likely to suffer from macro and micronutrient deficiencies because they increased demands of pregnancy and lactation. Furthermore, underweight mothers need to gain more body weight during pregnancy to fulfil the increased demand for nutrients during pregnancy and lactation. Underweight mothers were 3.3 times more likely to have a stunted child when compared with normal weight mothers. Our finding is similar to a 3.4 higher odds of stunting of children born from underweight mothers in Ethiopia [33] and lower than the 4.45 reported from Bangladesh [44]. Further analysis of the effect of BMI on stunting found that for every unit increase in the BMI scale the odds of stunting were reduced by 4% in Ethiopia (Table 3). A similar analysis in India reported that a unit increase in BMI reduced it by 3% [45]. The BMI as a scale is very general and is unable to differentiate between tall and thin BMI versus short and heavy body stature. Because stunting is a height for age measurement, it is important to consider the effect of maternal height in addition to her BMI. Taller mothers are more likely to have less stunted children that shorter mothers. The results of this study revealed that maternal height is significantly associated with stunting. Mothers who were less than 150 cm had 2.5 times higher odds of stunting when compared with taller (above 160 cm) women. In Tanzania, reducing 1 cm of maternal height increased the odds of stunting by 12% [46]. Our study found that, increasing 1 cm of maternal height reduced the odds of stunting by 1% (*p* = 0.01). This finding appears to point to the intergenerational transmission of stunting where shorter mothers tend to have stunted children, who grow up to be short mothers and have stunted children.

### Age, antenatal and postnatal care

The age of the mother is an important determinant in the outcome of her pregnancy, especially in developing countries where child marriage is still a common practice. Delaying the age when mothers have their first child by one year was associated with a 9% decrease in the odds (OR = 0.909, *p* = 0.001) of stunting (Table 3) and this is higher than the 7% per year reduction reported in Kenya [47]. Analysis of the effect of antenatal care shows that the number of ANC visits before delivery and place of delivery were significantly associated with childhood stunting (Tables 3). Mothers who regularly visit ANC clinics are more likely to be living in urban areas, wealthy and/or educated. Therefore, care should be taken in the interpretation of these findings. Having at least 4 ANC visits decreases the odds of stunting by 24% when compared with no ANC visits (Table 3). According to the latest national survey, 32% mothers had at least 4 four ANC visits [16]. Similar to our findings, having less than 4 ANC visits was significantly associated with higher odds of childhood stunting in Tanzania [21] and Indonesia [32]. For every ANC visit a mother makes, the odds of stunting were lowered by 7% (Table 3).

The protective effect of having “enough” time between consecutive births has been published previous Ethiopian studies [20, 34, 48]. Our analysis found that delaying birth interval by one month resulted in a 1% reduction in the odds of childhood stunting (Table 3). Mothers who have access to family planning services and have enough autonomy to decide when to have a baby are probably living in urban areas, have some education and/or are economically better off than their counterparts. In 2016, 74% of Ethiopian women gave birth at home [16] and mothers who gave birth at home were 33% more likely to have a stunted child when compared with those who gave birth at a health facility (Table 3). A small sample size case-control study by Kahssay found the place of delivery not to be a significant predictor of stunting in Ethiopia [34]. Home delivery was associated with a 24% increased odds of stunting in Tanzania [21] and 65% increase in Libya [26]. Women who deliver in a health facility are more likely to get prenatal and postnatal services, medical help for childbirth complications, vaccinations, and possibly nutritional counseling. Thus, Ethiopia needs to work on increasing health facility access and encourage pregnant women to follow ANC services and deliver in health facilities.

In a country where only 2% of women complete primary school and 4% have completed secondary or above [16], there is an urgent need to focus on girls’ education. If the protective effect of education is to bear any fruit, advocating for primary education for girls is not sufficient and girls ought to be encouraged to pursue secondary or above education. This paper is based on data from the 2016 EDHS which is a cross-sectional study. Thus, there are some limitations associated with such types of studies. First, it is difficult to determine causation from cross-sectional studies. Secondly, the DHS uses self-reported information which is subject to recall bias. Furthermore, the measurement of maternal autonomy did not include information about when the respondent started to work which means the duration of employment was not clearly defined. Joint decision making by both partners does not necessarily mean that the woman was given equal decision-making.

## Conclusion

This study showed that maternal educational level, maternal autonomy, maternal height, and BMI, number of antenatal care clinic (ANC) visits, and delivery in a health facility were significant predictors of stunting. Increasing maternal educational level reduced the odds of childhood stunting by 6.8% for every year a girl spends in school. Underweight and short stature mothers had higher odds of stunting when compared to their normal weight and taller counterparts. And mothers delivering in health facilities had 23% lower odds of stunting and underweight mothers were 2.4 more likely to give birth to a stunted child. Therefore, prioritizing the education of girls and improving their access to reproductive health services should be included in the policies aiming to combat stunting in Ethiopia.

## Data Availability

The DHS MEASURE general datasets used in this study was provided by the DHS Program available online at (https://dhsprogram.com/data/available-datasets.cfm).

## Abbreviations

ANC: antenatal care
BMI: body mass index
DHS: Demographic Health Survey
EDHS: Ethiopian Demographic
HAZ score: Height-for-age Z score
LRT: likelihood ratio test
SD: Standard Deviation
SSA: Sub-Saharan Africa
